# Effect of linaclotide combined with polyethylene glycol on bowel preparation before colonoscopy in patients with constipation

**DOI:** 10.3389/fmed.2026.1686654

**Published:** 2026-01-23

**Authors:** Lulu Liu, Jie Zeng, Jue Wang, Yan Zheng, Haiyan Liu, Zhipeng Liu, Ruiyu Wang, Sai Gu

**Affiliations:** 1 Department of Gastroenterology, The First Affiliated Hospital of Chongqing Medical University, Chongqing, China; 2 Emergency Medicine Center, Sichuan Provincial People’s Hospital, University of Electronic Science and Technology of China, Chengdu, China

**Keywords:** bowel preparation, colonoscopy, constipation, linaclotide, polyethylene glycol

## Abstract

**Background:**

Linaclotide, a promising medication for treating irritable bowel syndrome with predominant constipation, has the potential to enhance bowel preparation for colonoscopy. This study aims to evaluate the efficacy and tolerability of linaclotide combined with low-volume polyethylene glycol (PEG) in constipated patients.

**Methods:**

In this randomized, single-blind controlled trial, 210 constipated patients were assigned to one of three groups: (1) 3 L PEG (standard regimen), (2) 2-day linaclotide + 2 L PEG (2D + 2 L PEG), or (3) 4-day linaclotide + 2 L PEG (4D + 2 L PEG). Bowel cleansing was assessed using the Boston Bowel Preparation Scale (BBPS). Adverse symptoms, detection rates of intestinal findings, patient satisfaction, and willingness to repeat the procedure were recorded.

**Results:**

No significant differences in overall BBPS scores or detection rates were observed among the three groups. However, the 4D + 2 L PEG group showed superior left colon cleansing compared to the 2D + 2 L PEG group. Both linaclotide groups experienced fewer adverse symptoms (bloating, nausea, and vomiting) and reported higher satisfaction than the 3 L PEG group.

**Conclusion:**

A four-day regimen of oral linaclotide combined with 2 L PEG offers effective and well-tolerated bowel preparation with fewer adverse symptoms, making it a favorable option for constipated patients undergoing colonoscopy.

**Clinical Trial registration:**

http://www.chictr.org.cn (registration no. ChiCTR2400083515).

## 1 Introduction

The quality of bowel preparation is a critical factor influencing the effectiveness of colonoscopy. Inadequate bowel preparation may lead to misdiagnosis and subsequent poor clinical outcomes (1). Despite its importance, approximately 18–35% of patients fail to achieve adequate bowel preparation prior to colonoscopy (2). This issue is particularly pronounced in individuals with constipation, who often exhibit reduced colonic motility and drier stool consistency, thereby limiting the effectiveness of standard preparatory regimens (3).

Polyethylene glycol (PEG) has been widely utilized as the standard bowel cleansing agent in clinical practice (4, 5). However, the large volume required in conventional protocols (3–4 L) poses a significant barrier to patient adherence, often leading to poor tolerability due to bloating, nausea, and abdominal fullness (2). Particularly for specific clinical populations, such as the elderly and patients with cardiorenal comorbidities, high-volume fluid intake may be contraindicated or poorly tolerated. Previous studies have demonstrated that reducing the volume of PEG while adding adjunctive medications (such as bisacodyl and lubiprostone) can mitigate adverse effects without compromising the effectiveness of bowel preparation (6–8).

Linaclotide, a guanylate cyclase-C (GC-C) agonist approved by the U.S. Food and Drug Administration, has demonstrated efficacy in promoting intestinal fluid secretion and stool hydration, thereby enhancing defecation frequency. It is primarily indicated for the treatment of chronic idiopathic constipation and constipation-predominant irritable bowel syndrome (9, 10). However, its potential role as an adjunct to low-volume PEG for bowel preparation prior to colonoscopy has not yet been explored.

This study aims to evaluate the efficacy and safety of linaclotide in combination with low dose of PEG for bowel preparation in constipated patients, thereby providing a novel strategy for bowel preparation in patients with constipation.

## 2 Materials and methods

### 2.1 Participants

All participants in this study were outpatients recruited from the Gastroenterology Clinics of the First Affiliated Hospital of Chongqing Medical University. The sample size was calculated using a two-sided significance level (*α*) of 0.05 and a power (*β*) of 80%. The study was conducted from April 29, 2024, to June 30, 2024. Eligible participants included patients aged 18–85 years who were diagnosed with constipation and scheduled for colonoscopy. Constipation was diagnosed according to the Rome IV criteria.

Participants were excluded if they met any of the following criteria: (1) age under 18 years; (2) severe cardiac, hepatic or renal dysfunction; (3) severely active inflammatory bowel disease, severe gastroparesis, gastrointestinal obstruction or perforation, or toxic megacolon; (4) suspected or diagnosed psychological disorders; (5) use of medications that may cause coagulation disorders; (6) use of medications that may induce constipation, diarrhea, or other gastrointestinal dysmotility; (7) Use of linaclotide within 7 days prior to enrollment or known allergy to linaclotide; (8) pregnant or lactation during the study period; (9) refusal to provide written informed consent; (10) allergy to foods or drugs used for colonoscopy preparation.

The elimination criteria were as follows: (1) non-compliance with linaclotide administration requirements; (2) failure to complete bowel preparation or colonoscopy.

Informed consent was obtained from all participants, ensuring voluntary participation and full understanding of the study details. The study protocol was approved by the Ethics Committee of the First Affiliated Hospital of Chongqing Medical University (no. 2022104) and registered on April 26, 2024, at http://www.chictr.org.cn (registration no. ChiCTR2400083515).

### 2.2 Study design

This study was a randomized, single-blind controlled trial. A research nurse used SPSS (version 22.0) to generate a randomization table. Eligible participants were randomly assigned in a 1:1:1 ratio to one of three groups: the 3 L PEG group, the 2D + 2 L PEG group, and the 4D + 2 L PEG group. All participants followed a standardized low-fiber diet until the day before colonoscopy.

In the 3 L PEG group, participants received 3 L of PEG in a split-dose fashion. Specifically, 1 L PEG was administered at 8:00 p.m. on the day before the bowel examination, followed by 2 L of PEG 6 h before colonoscopy. In the 2D + 2 L PEG group, participants received 290 ug of linaclotide daily for 2 consecutive days before colonoscopy, and 2 L of PEG were administered 6 h before the procedure. In the 4D + 2 L PEG group: participants received 290 ug of linaclotide daily for 4 consecutive days before colonoscopy, along with 2 L of PEG administered 6 h before the procedure. To optimize bowel preparation, all participants received 30 mL of simethicone 30–60 min after completing their laxative regimen. Research nurses provided comprehensive instructions on dietary restrictions and laxative administration through both verbal explanations and written materials.

### 2.3 Primary outcomes

The primary outcome was the achievement of adequate bowel preparation, assessed using the Boston Bowel Preparation Scale (BBPS) (11). The BBPS evaluates bowel preparation quality by dividing the colon into three distinct segments (right colon, transverse colon, and left colon), with each segment scored individually. These segment scores are then aggregated to yield a total BBPS score ranging from 0 to 9. In this study, adequate bowel preparation was defined as achieving a score of 2 or 3 points in each colon segment or a total BBPS score of ≥6 (12, 13). All colonoscopies were performed by three highly experienced endoscopists (each with over 15,000 procedures), who were blinded to group allocation. Colonoscopy was considered complete when the colonoscope reached the cecum, with a minimum withdrawal time of 6 min to ensure adequate mucosal inspection.

### 2.4 Secondary outcomes

Secondary outcomes included detection rates of colon findings, patient acceptability, and adverse events. During colonoscopy, all detected polyps and adenomas were removed using standard forceps or a polypectomy snare and sent to the Department of Pathology for histological examination. Biopsies were taken when adenocarcinoma or inflammatory bowel disease was suspected.

Patient acceptability of bowel preparation (categorized as unacceptable, barely acceptable, or acceptable) and willingness to repeat the same regimen were assessed via questionnaires. Adverse symptoms during laxative intake such as bloating, abdominal pain, nausea, vomiting, fatigue, and hunger, were also recorded.

### 2.5 Data collection

Before the endoscopic examination, all the participants completed comprehensive questionnaires and underwent structured interviews conducted by a researcher. Detailed demographic and clinical data, including age, sex, height, weight, medical history of chronic diseases (specifically diabetes mellitus, hypertension, and coronary heart disease), previous abdominal surgeries, and prior colonoscopy experiences, were collected. After colonoscopy, both the colonoscopy reports with BBPS scores and endoscopic findings, as well as histopathological results from biopsies (including classification of polyps, adenomas, adenocarcinomas, and other relevant diagnoses) were systematically recorded by an independent blinded researcher. Additionally, adverse symptoms during bowel preparation and responses to questionnaires regarding the tolerability of the bowel preparation process and participants’ willingness to undergo repeat examinations were recorded.

### 2.6 Statistical analysis

Statistical analyses were performed using SPSS 27.0 (IBM Corp., Armonk, NY, USA). Continuous variables were expressed as median with interquartile range (IQR), while categorical variables were presented as absolute frequencies with corresponding percentages. The normality of continuous variables was assessed using the Shapiro–Wilk test, and the homogeneity of variance was evaluated using Levene’s test. For comparisons among the three groups, categorical variables were analyzed using the Chi-square (*χ*^2^) test. Quantitative variables were compared using the Kruskal-Wallis test when the assumptions of normal distribution or homogeneity of variance were not met. Afterward, pairwise comparisons were performed using the Mann–Whitney U test. A two-tailed *p* < 0.05 was considered statistically significant for all analyses.

## 3 Results

### 3.1 Baseline characteristics

**Figure 1:**
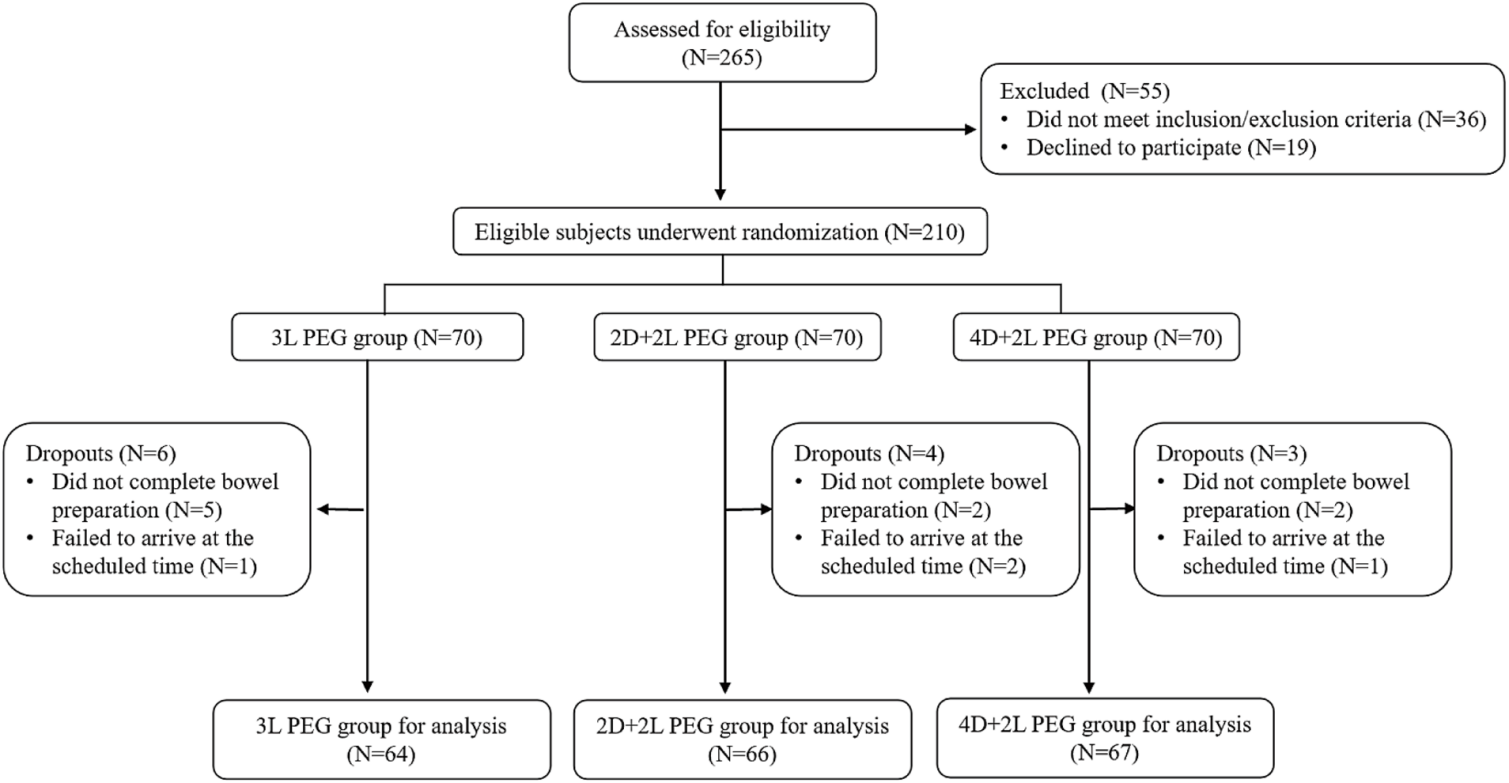
The study flow diagram.

The flowchart of participant recruitment is shown in Figure 1. Initially, 265 patients were screened for eligibility. Of these, 210 patients who met the inclusion and exclusion criteria were randomly assigned to three groups. During the study, 6 patients in the 3 L PEG group, 4 in the 2D + 2 L PEG group, and 3 in the 4D + 2 L PEG group withdrew due to various reasons, such as failure to complete bowel preparation or missed appointments. Ultimately, 197 patients completed the study and were included in the final analysis: 64 in the 3 L PEG group, 66 in the 2D + 2 L PEG group, and 67 in the 4D + 2 L PEG group. Baseline characteristics including age, gender, BMI, history of chronic diseases, and previous abdominal surgeries were comparable across the three groups (Table 1).

**Table 1:** Comparison of baseline characteristics.

Baseline demographics	3 L PEG (*N* = 64)	2D + 2 L PEG (*N* = 66)	4D + 2 L PEG (*N* = 67)	*p* value
Age, median (IQR)	61 (29)	60 (31)	56 (31)	0.569
Sex (female), *n* (%)	41 (64.1)	43 (65.2)	42(62.7)	0.957
BMI, median (IQR)	23.5 (5)	24.7 (6.02)	25.3 (7)	0.337
Diabetes, *n* (%)	17 (26.6)	15 (22.7)	13 (19.4)	0.621
Hypertension, *n* (%)	25 (43.1)	26 (39.4)	28 (41.8)	0.941
Coronary heart disease, *n* (%)	13 (20.3)	12 (18.2)	13 (19.4)	0.953
History of colonoscopy, *n* (%)	19 (29.7)	17 (25.8)	21 (31.3)	0.767
Abdominal operation, *n* (%)	6 (9.4)	7 (10.6)	6 (9.0)	0.956

Baseline clinical and demographic characteristics were collected from patients undergoing different bowel preparation regimens in three groups. IQR, interquartile range; BMI, body mass index.

### 3.2 Efficacy of bowel preparation

Bowel preparation efficacy was assessed using the BBPS score. Analysis of total BBPS scores revealed no statistically significant differences among the three groups: 6.91 ± 1.00 in the 3 L PEG group, 7.00 ± 0.99 in the 2D + 2 L PEG group, and 7.13 ± 1.01 in the 4D + 2 L PEG group (*p* = 0.457). Similarly, no significant differences were observed in the right colon scores (1.83 ± 0.49 vs. 1.92 ± 0.44 vs. 1.87 ± 0.63, *p* = 0.538) or transverse colon scores (2.50 ± 0.50 vs. 2.59 ± 0.50 vs. 2.49 ± 0.50, *p* = 0.454) across the groups.

Interestingly, the left colon BBPS scores were significantly higher in the 4D + 2 L PEG group compared to both the 3 L PEG group (*p* = 0.011) and the 2D + 2 L PEG group (*p* < 0.001), indicating superior bowel cleansing in this segment. No significant difference was found between the 2D + 2 L PEG and 3 L PEG groups (*p* = 0.289) (Table 2).

**Table 2:** Efficacy of bowel preparation.

BBPS score	3 L PEG (*N* = 64)	2D + 2 L PEG (*N* = 66)	4D + 2 L PEG (*N* = 67)	*p* value
Left colon	2.58 ± 0.50	2.48 ± 0.50	2.78 ± 0.45*^,#^	<0.001
Transverse colon	2.50 ± 0.50	2.59 ± 0.50	2.49 ± 0.50	0.454
Right colon	1.83 ± 0.49	1.92 ± 0.44	1.87 ± 0.63	0.538
Total	6.91 ± 1.00	7.00 ± 0.99	7.13 ± 1.01	0.457

The efficacy of bowel preparation was assessed by segmental and total BBPS (Boston Bowel Preparation Scale) scores among the three groups. ^*^p < 0.05 compared with the 3 L PEG group, ^#^*p* < 0.05 compared with the 2D + 2 L PEG group.

Detection rates of pathological findings including polyps, adenomas, adenocarcinomas, colitis, inflammatory bowel disease, and diverticula did not differ significantly among the three groups (*p* > 0.05) (Table 3).

**Table 3:** Colonoscopy findings.

Detection rate of lesions, *n* (%)	3 L PEG (*n* = 64)	2D + 2 L PEG (*N* = 66)	4D + 2 L PEG (*N* = 67)	*p* value
Polyps	24 (37.5)	27 (40.9)	28 (41.8)	0.87
Adenomas	8 (12.5)	7 (10.6)	6 (9.0)	0.806
Adenocarcinomas	1 (1.6)	2 (3.0)	1 (1.5)	0.779
Colitis	13 (20.3)	9 (13.6)	9 (13.4)	0.473
Inflammatory bowel disease	0 (0.0)	0 (0.0)	1 (1.5)	0.377
Diverticula	1 (1.6)	0 (0.0)	1 (1.5)	0.601

The detection rates of pathological findings, such as polyps, adenomas, adenocarcinomas, colitis, inflammatory bowel disease, and diverticula, were collected during colonoscopy among the three groups.

### 3.3 Acceptability and adverse symptoms

Acceptability of the bowel preparation regimen was evaluated based on participants’ acceptance of the dietary protocol and their willingness to undergo colonoscopy using the same method. Patients in both the 2D + 2 L PEG and 4D + 2 L PEG groups reported greater acceptance of the dietary regimen compared to those in the 3 L PEG group (both *p* < 0.05). Similarly, willingness to repeat colonoscopy with the same preparation method was significantly higher in the linaclotide groups than in the 3 L PEG group (both *p* < 0.001) (Table 4).

**Table 4:** Acceptability of participants.

Accessibility evaluation	3 L PEG (*N* = 64) *n* (%)	2D + 2 L PEG (*N* = 66) *n* (%)	4D + 2 L PEG (*N* = 67) *n* (%)	*p* value
Acceptance of diet plan				
Unacceptable	18 (28.1)	7 (10.6)*	6 (9.0) **	0.021
Barely acceptable	33 (51.6)	39 (59.1)	40 (59.7)	
Acceptable	13 (20.3)	20 (30.3)	21 (31.3)	
Willingness to repeat with the same bowel preparation	38 (59.4)	54 (81.8)**	53 (79.1)**	0.007

Acceptability of the bowel preparation regimen was evaluated based on the participants’ acceptance of the dietary protocol and their willingness to undergo colonoscopy using the same method. ^*^p < 0.05 compared with the 3 L PEG group; ^**^p < 0.001 compared with the 3 L PEG group.

Adverse symptoms during and after bowel preparation included bloating, abdominal pain, nausea, vomiting, fatigue, and hunger. Nausea was the most frequently reported symptom across all groups. However, the incidence of bloating and nausea was significantly lower in the 2D + 2 L PEG and 4D + 2 L PEG groups compared to the 3 L PEG group (bloating: 50% vs. 22.7% vs. 23.9%, p < 0.001; nausea: 53.1% vs. 30.3% vs. 26.9%, *p* = 0.003). No significant differences were observed among the groups for other symptoms (Table 5). Importantly, no episodes of urgency, fecal incontinence or other serious adverse events occurred in any group.

**Table 5:** Adverse symptoms.

Adverse symptoms	3 L PEG (*N* = 64) *n* (%)	2D + 2 L PEG (*N* = 66) *n* (%)	4D + 2 L PEG (*N* = 67) *n* (%)	*p* value
Bloating	32 (50.0)	15 (22.7)*	16 (23.9)*	<0.001
Abdominal pain	6 (9.4)	6 (9.1)	8 (11.9)	0.836
Nausea	34 (53.1)	20 (30.3)*	18 (26.9)*	0.003
Vomiting	25 (39.1)	15 (22.7)	15 (22.4)	0.054
Fatigue	16 (25.0)	21 (31.8)	22 (32.8)	0.57
Hunger	38 (59.4)	40 (60.6)	45 (67.2)	0.61

Adverse symptoms during and after bowel preparation included bloating, abdominal pain, nausea, vomiting, fatigue, and hunger were collected among the three groups. ^*^p < 0.05 compared with the 3 L PEG group.

## 4 Discussion

Colorectal cancer (CRC) remains the third most commonly diagnosed cancer globally, with its incidence and mortality rates rising significantly in Asia. In 2018, Asia accounted for the highest proportions of both incidence (51.8%) and mortality (52.4%) of CRC cases per 100,000 population worldwide (14, 15). Given the importance of early detection of intestinal tumors and adenomatous polyps, the quality of bowel preparation plays a decisive role in the effectiveness of colonoscopy (16). Therefore, selecting bowel preparation regimens that are safe, highly effective, minimally adverse, and well-tolerated is of paramount importance.

PEG is currently the most widely used solution for routine bowel preparation (4, 17). In most cases, a 4 L PEG regimen is commonly recommended to achieve optimal bowel preparation (18). However, due to the large volume and unpleasant taste, approximately 5–15% of patients are unable to complete the preparation process (19, 20). Although Chinese guidelines recommend a split-dose 3 L PEG regimen, many patients still experience discomfort during the procedure (21–23). Geriatric patients or those with impaired heart, kidney or liver function may even be incapable of taking 3 L PEG. Studies have demonstrated that chronic constipation is an important independent risk factor for inadequate bowel preparation (24). Various clinical conditions, including dementia, Parkinson’s disease, dehydration, and hypothyroidism, as well as medications, such as opiate analgesics, anticholinergics, diuretics, calcium channel blockers, anti-parkinsonian drugs, and oral iron supplements, are associated with constipation (25), which poses additional challenges in selecting regimens that ensure both efficacy and tolerability.

Previous studies have reported low-volume PEG-based preparations combined with various excipients can provide high rates of adequate bowel cleansing. For example, as a stimulant laxative, Bisacodyl is broken down in the large intestine after ingestion, and it directly stimulates intestinal peristalsis to facilitate defecation. A randomized controlled trial involving 400 patients with chronic constipation demonstrated that the combination of 2 L PEG and bisacodyl can achieve comparable bowel cleansing efficacy to 4 L PEG alone, along with better patient acceptance and compliance (6). In addition, a meta-analysis of 11 randomized controlled trials indicated that 2 L PEG combined with ascorbic acid showed no significant difference in bowel cleansing effectiveness compared to 4 L PEG alone (26). However, some studies have suggested that the PEG-ascorbic acid combination may increase the risk of acute kidney injury and electrolyte disturbances (27). Lubiprostone, often used for chronic constipation, has been found to improve the quality of bowel cleansing and allow for a reduction in the required PEG dosage (7, 8). Meanwhile, a recent clinical study reported that the addition of lubiprostone to PEG did not provide additional benefits for bowel preparation in chronic constipation patients undergoing colonoscopy (28). Therefore, it is of great significance to explore bowel preparation regimens that incorporate other excipients, with the goal of achieving satisfactory efficacy, improved safety, and enhanced patient compliance and satisfaction.

Linaclotide is a guanylate cyclase-C (GC-C) agonist that binds to GC-C receptors on intestinal epithelial cells, promoting chloride channel activation and increasing intracellular cyclic guanosine monophosphate (cGMP) levels (29). This enhances fluid secretion into the intestinal lumen, accelerates transit, and increases fecal water content and defecation frequency. Its pharmacological activity is largely confined to the gastrointestinal tract, with minimal systemic absorption and few systemic adverse reactions (30). Linaclotide has been widely used in the treatment of chronic constipation due to its favorable safety profile; moreover, it can also be effectively combined with other medications for patients with refractory constipation (10, 31). Additionally, it has been reported that linaclotide can serve as an adjunctive medication for capsule endoscopy and colonoscopy (32–35). However, limited studies have investigated its efficacy as a bowel preparation agent in patients with constipation.

Recent studies have demonstrated the efficacy of combining PEG with linaclotide for bowel preparation in patients with chronic constipation undergoing colonoscopy. A Chinese study involving 520 patients reported that 290 μg of linaclotide administered for 1 day with 4 L split-dose PEG, or for 3 days with 3 L split-dose PEG, yielded superior outcomes compared to the 4 L split-dose PEG group, without significant differences in polyp or adenoma detection rates (36). Similar findings were observed in another clinical trial (37), which enrolled 322 participants and reported that the 3 L PEG plus 870 μg linaclotide group (administered as a single dose for 3 days) resulted in significantly higher rates of adequate bowel preparation, better tolerance, and improved sleep quality compared to the 4 L PEG group. Building on this evidence, our study aimed to evaluate whether linaclotide could enable a further reduction in PEG volume (to 2 L) while maintaining efficacy.

Our primary findings demonstrate that linaclotide combined with only 2 L PEG provides favorable bowel preparation quality and diagnostic yield when compared with the standard 3 L PEG regimen. These results confirm that linaclotide can effectively compensate for a 33% reduction in PEG volume without compromising cleansing efficacy in patients with constipation. Importantly, both linaclotide groups (2-day and 4-day) reported significantly fewer adverse symptoms such as bloating, nausea, and vomiting, along with higher acceptance of the dietary regimen and greater willingness to repeat the preparation.

In the present study, another important finding was the superior cleansing of the left colon observed in the 4-day linaclotide group compared with both the 2-day linaclotide and the 3 L PEG groups. The left-sided colon, encompassing the descending colon, sigmoid colon, and rectum, is a common site for colorectal cancer, precancerous lesions and ulcerative colitis (UC) (38–40). Anatomical features such as a narrower lumen and stool stasis make the left colon more difficult to cleanse. Iacucci et al. reported that incomplete cleaning may conceal mucosal erosion, ulcers, or pseudopolyps in UC patients, potentially leading to misdiagnosis (41). Inadequate cleansing of the left colon increases the risk of missing adenomas and carcinomas, and poor preparation in this region has been associated with a measurable decrease in adenoma detection rates (42, 43). Thus, the enhanced left colon cleansing achieved with the 4-day linaclotide regimen is clinically meaningful. Several mechanisms may explain the observed differences in BBPS scores between the left and right colon. First, as a GC-C receptor agonist, linaclotide enhances fluid secretion and accelerates transit, making the contents of the left colon more liquid and easier to evacuate through propulsive peristalsis. In addition, the frequent defecation induced by linaclotide provides direct and thorough flushing of the left colon. By contrast, residues in the right colon tend to adhere to the mucosal wall after water absorption during earlier evacuations. Furthermore, patients with constipation often exhibit impaired colonic motility, with dysfunction being more pronounced in the right colon, thereby exacerbating the discrepancy in cleanliness between the two regions.

Compliance and acceptance are critical determinants of successful bowel preparation. The standard requirement of 4 L or 3 L of fluid intake often results in poor compliance or incomplete preparation for many patients. In our study, patients receiving linaclotide required only 2 L of PEG solution, which markedly improved both acceptance and willingness to repeat the regimen. This low-volume, linaclotide-augmented approach may provide an excellent option not only for constipated patients, but also for patients unable to tolerate large fluid volumes due to cardiovascular or renal comorbidities.

Linaclotide has been reported to induce ion secretion, thereby generating a transient hyperosmotic environment within the intestinal lumen that drives secretory diarrhea (30). Since a hypotonic environment does not physiologically induce diarrhea, linaclotide administration is more likely to result in hypertonic diarrhea. Hypertonic diarrhea is known to carry a risk of dehydration. Although no electrolyte disturbances or dehydration were observed in the combination group in our study, further confirmation is warranted. In future research, we plan to elucidate the underlying mechanisms by measuring fecal osmotic pressure and electrolyte concentrations across all groups.

## 5 Conclusion

This study demonstrated that a 4-day regimen of oral linaclotide combined with 2 L PEG achieves comparable bowel cleansing efficacy to 3 L PEG alone in constipated patients, with superior cleanliness in the left-sided colon. Additionally, this regimen significantly reduces adverse effects, enhances patient satisfaction and tolerability, and improves overall compliance.

## Data Availability

The raw data supporting the conclusions of this article will be made available by the authors, without undue reservation.
